# Lessons learned from approaches in COVID-19 risk Communication and community engagement in Malawi: an exploratory study

**DOI:** 10.1080/17482631.2025.2516356

**Published:** 2025-06-12

**Authors:** Chiyembekezo Focus Maganga, Lusizi Kambalame, Aeron M. A. Nahuku, Victor Lordwin Chikoti

**Affiliations:** a The Language and Communication Department, Malawi University of Business and Applied Sciences, Blantyre, Malawi; b Biomedical Sciences Department, Kamuzu University of Health Sciences, Blantyre, Malawi

**Keywords:** COVID-19, risk communication, community engagement, approaches, Malawi

## Abstract

**Purpose:**

During public health emergencies like COVID-19, approaches used in Risk Communication and Community Engagement (RCCE) are critical in the process of transmitting and exchanging health information. However, there is generally limited literature on COVID-19 RCCE approaches, particularly their strengths and limitations. The current study seeks to contribute to that literature by exploring approaches that were used in COVID-19 RCCE in Malawi, a sub-Saharan African (SSA) country, and drawing lessons from the response based on experiences of those who implemented it.

**Method:**

Using in-depth interviews with ten key informants and ten project reports from three organizations, data were analysed thematically in MAXQDA 2022.

**Results:**

Significant themes that emerged revealed COVID-19 RCCE employed use of interpersonal approaches like training, peer education, mHealth and social media; use of community mobilization strategies like engagement meetings and dialogues, loud hearing through mobile vans and whistle stops; behaviour change printed materials; and the mass media. Effectiveness hinged on each method’s accessibility, intrinsic qualities, and reach. Implementers also emphasized the value of combining multiple approaches and the challenges posed by COVID-19 restrictions.

**Conclusion:**

The study underscores the need to select context-appropriate, accessible RCCE approaches to convey critical information, and contributes to the evidence base for future pandemic responses, especially in resource-limited settings.

## Background

COVID-19 brought in a global public health crisis that affected every sphere of life. The World Health Organization (WHO), on 11 March 2020, declared COVID-19 a pandemic, becoming the first coronavirus disease to be characterized as such (WHO, 2020). One year into the outbreak, the disease pushed 70–100 million people into extreme poverty and created an information and socio-economic crisis (WHO, 2020).

During public health emergencies like COVID-19, the WHO recommends Risk Communication and Community Engagement (RCCE) as one of the key pillars in the strategic preparedness and response plan (WHO, 2020). The WHO African region also included risk communication, community engagement and infodemic management in its 11 strategic pillars of the 2021 COVID-19 Strategic Preparedness and Response Plan (COVID-19 SPRP) (WHO, 2021). Risk Communication and Community Engagement (RCCE) in the 2021 COVID-19 SPRP aimed to reduce the pandemic’s adverse impacts on individuals and communities by providing timely, credible and relevant information to contain the infodemic and ensure implementation of people-oriented and community-led approaches that can enhance trust and social cohesion. Further, RCCE is also said to enhance the propensity for resilience as communities are engaged and viewed as essential stakeholders in the response (Khan et al., 2022).

The importance of RCCE during health emergencies became heightened during the Ebola outbreak in West Africa when it was observed that providing correct and timely information, addressing panic and anxiety in both the public and frontline responders, and engaging communities in the response played an enormous role in the prevention and control of the disease (WHO, 2018). Further, advancements in new digital technologies and effective ways of communicating and engaging stakeholders during crises have globally been embraced as having significant prospects for addressing issues of public health concern and facilitating social and behavioural change (Fayoyin, 2016).

A growing evidence base from studies conducted on the effectiveness of various approaches in addressing public health issues shows that methods differ in their appropriateness based on the channels’ own intrinsic characteristics (Fayoyin, 2016; Mbuya et al., 2020). For example, a systematic review on the effectiveness of peer education interventions for HIV prevention in developing countries established that the approach was effective in increasing HIV knowledge and enhancing HIV preventive behaviours (Medley et al., 2009). However, the approach, which recruits individuals who share demographic characteristics or risk behaviours with a target group and equip them with knowledge and skills to change behaviour among members of the same group, was found to be ineffective in reducing new HIV infections (Medley et al., 2009).

Further, a study that evaluated the use of social media in health communication concluded social media and E-Health, which is referred to as the employment of electronic transmission or digital media to enhance health communication, pose opportunities for health promotion programmers and implementers to confront various health issues affecting the African continent (Fayoyin, 2016). The study stated social media platforms have features that can facilitate interactivity, user-generated content, horizontal communication, multi-directionality of messages, genuine dialogues and speed, all which strengthen the effectiveness of health messages carried through the approach. Mass media have also been found to have attributes that can influence behaviour change at an individual level and social change at a community level by raising public awareness and individual participation in health care issues (Al-Dmour et al., 2022).

Studies specific to RCCE approaches used in previous disease outbreaks examined different strategies and drew different conclusions. For example, a study investigating risk perception during the 2014–2015 Ebola epidemic in Sierra Leone concluded that active interaction between programme implementers and the target audience effectively addresses low-risk perceptions (Winters et al., 2020). In Ethiopia, the mass media, mainly the television and radio, were found to be the most broadly used approaches for transmitting Cholera messages, though television was the one more preferred (Adera et al., 2022).

Lessons, however, continue to build up on various approaches used in COVID-19 RCCE although gaps still remain in literature. Studies we found that investigated COVID-19 approaches either focused on one approach (Nsubuga et al., 2023) or did not examine the approaches comprehensively (Adebisi et al., 2021). The current study seeks to change that by investigating approaches used in COVID-19 RCCE in Malawi and the lessons implementers drew from implementing the RCCE response.

## Methods

### Design

The study employed an exploratory approach to understand the strengths and limitations of various strategies used in COVID-19 RCEE. This exploratory approach, which falls under qualitative research, allowed for an in-depth understanding and a better explanation of the subject matter (Skinner et al., 2015). The explanatory approach is also deemed effective for researching areas that are under-investigated in the health field (Knezevic et al., 2023).

### Study setting and participants

This study was conducted in Malawi, a country with a population of about 18 million people, whose above three-quarters of the population live in rural areas (84%) and practice Christianity (75%), and where slightly above two-thirds of the population (68%) are literate (National Statistical Office [NSO], 2019). Further, more men than women in Malawi have access to ICT facilities and digital technologies (NSO, 2020). Additionally, as of the time of this study, the country had 13 government hospitals, which include four central hospitals, and in early 2020, only the central hospitals were equipped to provide mechanical ventilation, of which the country had 16 ventilators in total (Mzumara et al., 2021).

In Malawi, the COVID-19 RCCE response was led by the country’s Ministry of Health (MoH) the health education section and involved several local and international organizations. Through Health Communication for Life (HC4L), USAID supported the MoH and local organizations to develop and implement Social and Behaviour Change Communication (SBCC) interventions as part of the COVID-19 RCCE response. The response also included the implementation of a national COVID-19 Campaign, dubbed *Osayidelera COVID-19 Campaign*, which can loosely be translated as “don’t underestimate or underrate COVID-19”. The campaign aimed to increase knowledge of COVID-19, increase disease risk perceptions and enhance compliance with public health measures. The MoH and HC4L helped coordinate the campaign which was being implemented by several organizations.

This exploratory study was conducted in Malawi. Ten participants involved in the COVID-19 RCCE response were selected and interviewed. We also conducted ten project reviews of RCCE response from three organizations- Gaka FM, Communications Trust-DCT and Centre for Development Communications. The three organizations were selected purposefully because they were the ones that had already prepared comprehensive reports that covered areas that were relevant to the scope of the study. Once the organizations were selected, the study used purposive sampling again to choose a key person in each selected organization who was directly involved in facilitating or coordinating the COVID-19 RCCE response. In-depth interviews with the key informants were employed to understand approaches employed in COVID-19 RCCE and their strengths and limitations based on the experience of the key informants. Table 1 summarizes the socio-demographic characteristics of participants.

**Table 1.** Descriptive summary of participants’ characteristics.

Serial Number	Demographic Characteristics		Frequency of Participants (*n* = 10)
1	Age	30–40	6
		41–50	4
2	Gender	Male	8
		Female	2
3	Role in RCCE	Supervisory	4
		Coordination	6
4	Sector	Government	3
		Non-governmental	7

### Data collection procedure and tools

A practicing journalist who was also a master’s student in Health and Behaviour Change Communication supported this study with data collection. The journalist was selected based on his interview experience and interest in the subject matter. The first author trained the journalist on the study’s objectives and the required data depth. The interviews lasted approximately 20 min. The interview guide covered participants’ demographic factors like age, sex and role in RCCE response; The RCCE approaches that were used and why the approaches were selected in the organization- including which methods worked or failed; challenges faced in the implementation; and recommendations. The interviews were conducted virtually and audio-recorded due to COVID-19 restrictions during the discussions. Additionally, we reviewed project reports from Gaka FM radio station, Communications Trust-DCT and Centre for Development Communications. The organizations this study collected project reports from were selected purposefully based on richness (depth) and relevance of data in their reports. All the reviewed reports were in text format. In the current study, we checked from the reports data on approaches, activities or strategies and lessons drawn in the response.

### Data analysis

Audio data from the in-depth interviews were transcribed and, together with relevant sections of RCCE reports, entered into MAXQDA 2022 software to help systematically organize, code and structure the data for easy analysis. Thematic analysis was employed to analyse the data. To guarantee reliable coding and thematic identification, check coding was used. This is a process by which researchers independently code initial data collected and reconcile differences and disagreements. After the individual work, a consensus-forming process was used to identify overarching themes o r patterns within the data. Data from in-depth interviews and reports were merged during data analysis, and all the authors were involved in data analysis. For reliable coding and theme identification, we employed the following steps of doing thematic analysis as described by Maguire and Delahunt (Maguire & Delahunt, 2017): 1) become familiar with the data; 2) generate initial codes; 3) search for themes; 4) review themes; 5) define themes; and 6) writing-up.

### Ethical considerations

The study was part of an evaluation of the Osayidelera COVID-19 Campaign implemented in Malawi as part of the country’s COVID-19 RCCE response. The National Health Sciences Research Committee based in Malawi granted the ethical approval to carry out the study. The study sought verbal approvals from respective organizations that were part of the study. The participants signed informed consent forms upon reading the information sheet and agreeing to participate in the study. The participants agreed to participate without receiving any compensation for their time.

## Results

### Demographic characteristics

This study reviewed ten RCCE progress reports from three organizations and conducted intensive interviews with ten participants. Most of the participants were males (8), aged 30–40 (6), played a coordinating role (6) and came from nongovernmental organizations (7%), as is summarized in Table 1.

### Findings

There were four overarching themes that emerged from our data on approaches that were used in COVID-19 RCCE response in Malawi- interpersonal social and behaviour change communication (SBCC), community mobilization, printed Social and Behaviour Change materials and the mass media. Other pieces had subthemes that also occurred. For lessons that were learnt from the response, four overarching themes also emerged: Intrinsic characteristics of the used approach matter, the reach of an approach is an important factor, COVID-19 restricted the use of face-to-face interactive channels and blending approaches help. Additional quotes for themes and subthemes are provided in Table 2.

**Table 2.** Major themes and subthemes that emerged from data and examples of their respective quotes.

Theme	Subthemes	Examples	Source
APPROACHES USED Interpersonal communication (IPC)	Training and workshops	Trainings to me was best approach that we manage to reach out to these people because during the training we could really see this people somehow, they had a lot of misinformation a lot of misconceptions so with that we were able to clear out those misconceptions, we were able to give them the right information that supposed to be going around telling people	P001
		People were having misconceptions to say health workers are killing people to get allowances, so we were trying to clear out those misconceptions, but we were doing that using training, like we trained all the HSAs because they go around giving the information so we trained them that when they are asked, they could give out correct information.	P001
	Peer education	I can single out that this was effective because they gave an opportunity to the one disseminating the information to interact with household members and, in the process, they could understand the issues in context and be able to give appropriate information.	P004
		The door-to-door campaign strategy is a practical and interactive type of community engagement. First, this approach helps to gauge the level of awareness the household members have about COVID-19. This helps to frame messages correctly to give comprehensive information on prevention measures or care seeking depending on the assessment of knowledge levels. Household members, in turn, can ask questions or seek clarification on issues requiring more information or clarification. The door-to-door meetings are effective as members can observe some behaviours/practices at the household level, which may contribute to the spread of COVID-19.	Report 2
	MHealth	so if someone is sick there were some hotlines numbers that were being used but there were national hotline numbers, so people would call those numbers seek for like assistance so I would like call for an ambulance to say I have a covid patient or a suspect so I need help”	P005
	Social media	We liked to use traditional media, but we also engaged some social media influencers so were also present on various social media platforms	P006
Community mobilization	Community Meetings and dialogues	We were able to share information and share plans and give each other updates and we could plan accordingly about who is doing what and where, we could see where the gaps are in terms of coverage, we could also see where there are duplications, but unfortunately, we only had few meetings.	P002
		Area Development committee (ADC) meetings have proved to be a local governance structure used as a channel of communicating messages to community members. This has also worked in disseminating COVID-19 messages by sensitizing local leaders and influential people about COVID-19 prevention messages, dispel myths and misconceptions on COVID-19, and promote care seeking and home-based management of COVID-19 symptoms.	Report 2
	van mobilizations and town/village criers	I could single out loud hearing. The loud hearing was simply informing people that there is Covid − 19, it wasn’t interactive.”	Male 5
		The van spread COVID-19, awareness and preventive messages in the community through speakers mounted on a vehicle. The van plays COVID-19 prevention jingles, music, PSAs and radio spots produced for the Osayidelera COVID-19 campaign as well as Community specific messages spoken out by DHPOs and HSAs in the district.	Report 1
		DCT uses a mobile van going through targeted villages and identified hotpot areas. The use of mobile van aims at reaching community members with COVID-19 messages without convening at one place where physical distancing may be a challenge. The whistle stop prioritize mapped hotspots such as trading centres, villages with conformed cases of COVID-19, border districts where cross border trade take place and targeting the general community. These whistle stops are effective because for instance at a busy trading centre people do get COVID-19 messages while selling or buying commodities. Our members conducted five (5) early morning and late evening village criers/announcements aiming at raising awareness on COVID-19 and prompt care seeking upon noticing signs of coronavirus.	Report 3 Report 7
	Mobile cinema and whistle stops	and where it was safe we also had what we call mobile cinema where we mounted screen where people can watch and we were very strategically on that because discovered that if people gather together and congested is also not safe so wherever we had this a cinema a mobile cinema we could put people, placed people at safe distance so that they cannot be a source of infection to one another	P004
SBCC printed materials	N/A	Basically, as I said when we go out have a meeting in the communities maybe we could just use posters which had messages and pictures.	
		I could say like the model that we used for distributing materials to minibus drivers, saloons and barbershops, I can’t say it failed, but it was difficult to monitor. Of course, we gave them feedback lines but still more it was difficult to monitor whether they were indeed playing the CDs.”	P008
Mass media	Radio Radio	unlike real phone in programs where you can get instant feedback and you can even tell the direction that you have been taking	P002
		use community radio stations because they are the ones who have knowledge about their community, they also have knowledge on how they can change behaviour of the people of that area	P007
	Radio Listening clubs	Under community mobilization, the project mobilized two Radio Listening Clubs, one in each district in the identified COVID-19 hot spot areas, whose members have undergone capacity building sessions to equip them with knowledge and skills on how to reach communities with correct information about COVID-19.	Report 5
	Radio phone-in	so we had programs in radio we introduced what we call radio phone in programs where we went into the radio with experts like from district health office, from social welfare office even from police depending on what topic we want people to hear about so they would talk to people on air and later open the airwaves	P010
**LESSONS LEANT**	Radio listening	Radio listening clubs were built capacities and given skills on how to reach community members with correct messages and dispel myths and misconceptions about COVID-19; increase risk perception and adoption of infection prevention practices/behaviours and promote prompt care seeking and home-based management of COVID-19 symptoms.	Report 6
Intrinsic characteristics of the approach matter	N/A	The targeted door-to-door approach provided an opportunity for communities to ask questions and get immediate response.	Report 2
The reach is an important factor	N/A	I think because most people listen to the radio, we mostly used the radio because it’s where we can reach people without any challenge.	P007
Covid-19 restricted face-to-face interactive channels	N/A	At time we could think of using theatre for development because theatre for development gives us an opportunity to unearth issues which are on the ground and communication through drama and other activities is contextualized. But in this case we couldn’t use that one because of the restrictions which were emanating from the nature of the disease itself, let’s say no gatherings.	P003
Blending approaches matter	N/A	Local leaders in Dedza and Machinga led by the Traditional Authorities Kachere and Nkula respectively are in the process of formulating community based by-laws to enforce measures that would address the expected behaviours. For instance, the by-laws would address case management whereby a conformed COVID-19 case would be under strict observation by family members to ensure isolation and quarantine measures are observed.	Report 1

### Approaches used in RCCE

#### The use of interpersonal approach

##### Using training and workshops

This approach was designed to enhance knowledge and skills among targeted stakeholders to gauge mis/disinformation, misconceptions and rumours about the pandemic. Participants revealed during training that they could tell the colossal information gaps and that many training and workshop attendees needed correct information about the pandemic. Some of the targeted groups for training were Health Surveillance Assistants (HSAs), community volunteers, radio listening clubs and journalists from community radio stations. Participants who used this approach said it effectively empowered disseminators of COVID-19 information with verifiable information about the pandemic.

One participant explained how it worked:

People had misconceptions to say health workers are killing people to get allowances, so we were trying to clear out those misconceptions, but we were doing that using training like we trained all the HSAs because they go around giving the information, so we trained them that when they are asked, they could give out correct information- P001.

##### Using peer-based approaches

The response also used peer approaches like peer education, which involved volunteers from community-based organizations or teachers as peer educators. It also used door-to-door visits by trained HSAs and radio listening clubs. Some participants considered this approach most effective, enabling facilitators to give information tailored to the context. For example, one of the reports explained:

… the door-to-door campaign strategy is an effective and interactive community engagement. First, this approach helps to gauge the level of awareness the household members have about COVID-19. This helps to correctly frame messages to give comprehensive information on prevention measures or care seeking depending on the assessment of knowledge levels. Household members, in turn, can ask questions or seek clarification on issues requiring more information or clarification. The door-to-door meetings are effective as members can observe some behaviours/practices at household level which may contribute to the spread of COVID-19- Report 2.

A respondent also explained:

Ones that I think that worked were door-to-door visits by community volunteers, these are members of community organisations, as well as door-to-door visits that teachers conducted. Those are the ones I can single out that were effective because they allowed the one disseminating the information to interact with household members. In the process, they could understand the issues in context and be able to give appropriate information- P005.

Some participants, however, explained that this approach was difficult to implement as communities resisted any information about COVID-19 and threatened anybody who came with information about the pandemic. Data from reports and participants also indicated restrictions on social gatherings and social distancing limited the implementation of the approach.

##### Using e-health

The reviewed reports and narratives from participants indicated e-Health or electronic communication were used in two ways: Mobile phone technology for health (mHealth) and social media. In the Malawian context, mHealth was employed using either traditional texts or a hotline to connect health specialists with patients or their guardians. Participants said the initiative broadened the horizon of the suspected and confirmed COVID-19 cases interacted with medical professionals. According to the participants, some people who shunned healthcare services at public health facilities could seek their medical advice through phones. One participant explained:

From our findings people were scared to go to hospital; instead, they were at home, but people could call DHO [district health office] and ask for assistance- P002.

Social media was said to have been widely used in response to community feedback or as a social listening tool. The participants said they used social media to receive community feedback about their COVID-19 activities. Further, they said they used social media influencers to help disseminate COVID-19 information on various platforms.

#### The use of community mobilization approach

##### Using engagement meetings and dialogues

This approach was used as part of formative research to inform the design of the RCCE response in targeted areas and as part of continuous engagement with stakeholders during the implementation of the reaction. As a report explained, this approach was effective in enhancing collaboration and adherence to COVID-19 preventive measures:

Area Development Committee (ADC) meetings have proved to be a local governance structure used to communicate messages to community members. This has also disseminated COVID-19 messages by sensitising local leaders and influential people about COVID-19 prevention messages, dispel myths and misconceptions on COVID-19, and promote care seeking and home-based management of COVID-19 symptoms-Report 2.

##### Loud hearing through mobile vans and town/village criers

Participants revealed they used mobile vans to disseminate COVID-19 awareness and preventive messages in the community through speakers mounted on a vehicle. The van played COVID-19 prevention jingles, music, Public Service Announcements (PSAs), the Osayidelera COVID-19 campaign radio spots, and Community-specific messages. Participants also explained they could use village/town criers who employed their natural voice, megaphones, or sometimes a mobile speaker on a bicycle or motor bicycle to spread COVID-19 preventive messages across targeted communities. Some participants applauded these approaches as a quick way to communicate with people across communities using their language. However, some participants also said the process was limited because it was not interactive.

I could single out loud hearing. The loud hearing informed people that there is COVID–19; it wasn’t interactive- P006.

##### Use of whistle stops or mobile cinemas

The participants who used this approach said it targeted mapped hotspots such as trading centres, villages with confirmed cases of COVID-19 or border districts where cross-border trade occurs. The idea was to reach out to targeted people at hotspots where they are found.

These whistle stops are effective because, for instance, at a busy trading centre, people get COVID-19 messages while selling or buying commodities-Report 3.

Further, the approach was coupled with question-and-answer sessions, which enabled people to understand the messages richly. However, since this approach attracted large audiences, participants explained that it was unsafe during the pandemic.

… because discovered that if people gather together and congested is also not safe so wherever we had this a cinema a mobile cinema, we could put people, placed people at a safe distance so that they cannot be a source of infection to one another-P004.

#### Use of social and behaviour change (SBC) printed materials

##### Blended with interpersonal approach

This approach which combined the use of printed SBC materials with interpersonal communication to provide implementers a platform to get some feedback. One participant explained the effectiveness of this approach.

*We were using interpersonal communication, we could also use print visuals such as posters and leaflets, and we were also using face-to-face channels to allow community members to ask questions- P005.*

##### To be pasted on strategic places

In this approach, the materials were pasted on strategic places or distributed to minibus drivers, salons, barbershops and other areas that attract people. Some participants said they found this approach effective in terms of reach as materials could be distributed to many people quickly and that printed messages are permanent. However, other participants said the effectiveness of this approach was difficult to determine since implementers were, in most cases, unable to get any feedback on whether the materials were read and used. However, a participant explained that the approach was designed to allow audiences to get feedback.

Of course, we gave them feedback lines, but still more it was difficult to monitor whether they were playing the CDs- *P007*.

#### The use of mass media

Most participants also said they used traditional mass media in their response. The radio, particularly community radio stations, was the mass media many organizations frequently used. The participants said community radio stations were deemed ideal because the stations know their audiences better and communicate with their target populations in the language understood by the audiences. Several organizations mentioned radio jingles as the form they mostly used to deliver COVID-19 messages on the radio. One participant narrated:

We used community radio stations because they are the ones who know their community; they also know how they can change the behaviour of the people of that area. We could localise the jingles to use-P002.

Participants also said they used radio listening clubs in targeted communities to do formative research to inform their RCCE programmes. The listening clubs could also be engaged to help in the design of jingles as a way of ensuring the message would be effective. To enhance the effectiveness of the messages, some participants explained that they opted for a phone-in programme to be able to get feedback.

Feedback could be hard to get except in real phone programs where you can get instant feedback, and you can even tell the direction you have been taking- P008.

### Lessons learnt from the response

#### Intrinsic characteristics of the media matter

Narratives from respondents and project reports highlight how intrinsic characteristics of an approach affect a strategy’s appropriateness and effectiveness. For example, it was revealed face-to-face strategies that facilitate two-way communication and which are more personal were deemed to be most appropriate when communicating complex issues about the disease as explained by a respondent:

Door-to-door visits, for example, that were conducted by teachers and other volunteers are the ones I can single out to be effective because they gave an opportunity to the one disseminating the information to interact with household members, and in the process they could understand the issues in context and be able to give their audience appropriate information- p009

On the other hand, respondents narrated the absence of feedback in approaches that are one-way made it difficult to assess the effectiveness of such channels. For example, a respondent explained:

I could single out loud hearing. The loud hearing was simply informing people that there is COVID − 19, it wasn’t interactive. And it was difficult to assess whether or not it is effective- p001.

Table 3 summarizes the strengths and limitations of various approaches used in COVID-19 RCCE) in Malawi. The assessment of these approaches was based on a consensus among the authors, who drew on data provided by implementers involved in the COVID-19 RCCE response, the theoretical robustness of each approach particularly as viewed in media richness, and the researchers’ practical experiences with these methods.

**Table 3.** A summary of the appropriateness of various approaches used in COVID-19.

Approach	Opportunity for multiple cues*	Chances and Speed of feedback*	Reach or penetration into community*	Feasibility during COVID-19*
1. Training and workshops	Highest	Highest	Moderate	Low
2. Peer education	Highest	Highest	Low	Moderate
3. MHealth	Lowest	Moderate	Moderate	Highest
4. Social media	High	High	High	Highest
5. Engagement meetings and dialogues	Highest	Highest	High	Low
6. Loud hearing through mobile vans and town/village criers	Moderate	Low	Very high	High
7. Use of whistle stops or mobile cinemas	High	High	Highest	Lowest
8. Social and behaviour change printed materials	Lowest	Lowest	High	Highest
9. Mass media (audio e.g radio)	Low	Generally low, but high on phone-in programmes	High	Highest
10. Mass media (visual e,g tv)	High	Generally low, but high on phone-in programmes	High	Highest

*Scale ranging from lowest to highest.

#### The reach of an approach is an important factor

The study revealed channels that are accessible to most people and which are able to penetrate deeply into communities are deemed more appropriate. In Malawi, several respondents described the radio as the most accessible channel in the country. For example, a respondent stated:

I think because most people listen to the radio, we mostly used the radio because it’s where we can reach people without any challenge.-p003.

#### Covid-19 restricted face-to-face interactive approaches

*Respondents and project reports revealed face-to-face interactive approaches, particularly those that attract large crowds were difficult to implement given restrictions on social gatherings and social distancing imposed during COVID-19. One respondent explained*:

Generally, face-to-face approaches that enabled two-way communication were effective in explaining complex issues about the disease. But you know with COVID-19, it meant activities that could attract large crowds were not allowed. That meant effectiveness of an approach was not the only factor to be considered in the response-p007.

#### Blending approaches help

Several respondents and project reports explained blending various approaches and using several channels on the same audience helped to enhance the reach and effectiveness of the response. For example, one project report explained:

Using multiple channels ensured that more people in the catchment area are reached. One could not miss the messages; if not from the town crier, it is the mobile van. If not the van then they will catch you at home through the door-to-door outreach. If not any of these then it is the radio programs or the posters, you surely get the messages one way or the other-CDC report 1.

One respondent also said:

My recommendation is that one size doesn’t fit all, we need to deploy a number of strategies so that the messages can reach as many people as possible.

## Discussion

The study sought to explore COVID-19 RCCE approaches in Malawi and to examine lessons learnt in implementing different approaches in the response. Our findings showed the use of four broad approaches namely: interpersonal, community mobilization, printed SBC materials and the mass media. They were subthemes like use of training, peer education and door-to-door visits, health, engagement, social media, meetings and dialogues, mobile vans, mobile cinema, social media and community radios that emerged from data. On lessons learnt by implementers, the study found intrinsic characteristics of the used approach matter, the reach of an approach is an important factor, COVID-19 restricted the use of face-to-face interactive channels and that blending approaches help.

Most of the approaches found in the study are similar to those found to have been used in Nigeria (Ogundijo et al., 2022), 13 African countries (Adebisi et al., 2021) and Singapore (Tam et al., 2021). The similarity could stem from frameworks informing the RCCE response (WHO, 2020) and the level of accessibility of the common approaches. The blending of several approaches found in the current study is also consistent with one of the major conclusions of a study in Nigeria that argued RCCE activities must not follow a “one-fits-all” approach but that they should combine various approaches and strategies (Ogundijo et al., 2022).

It is not surprising that data from the study revealed approaches that facilitated two-way communication like training and workshops, peer education, and community dialogues displayed supremacy in permitting the use of multiple cues and enabling immediate feedback and clarification as literature also shows such media have high richness (Lampa et al., 2021). Previous studies like those conducted in Uganda (Nsubuga et al., 2023), Malawi (Walsh et al., 2018) and the US (Rosen & Goodson, 2013) also demonstrated the effectiveness of engagements and dialogues in health interventions.

The importance of accessibility and trust, as they pertain to approaches used in health interventions, has also been highlighted in many studies, one of the most prominent one being a study that evaluated media and messages for nutrition and health in the Lao People’s Democratic Republic (Mbuya et al., 2020). The study found that the four most accessed approaches in communities were village announcements, mobile phones, television and out-of-home media, although the most trusted one was village announcements. While village announcements were also used in Malawi’s RCCE and most accessed at a community level, at a national and higher level, the radio is by far the most accessed channel in Malawi (NSO, 2019, 2020). The radio listening clubs were also successfully employed in other health interventions in Malawi (Nyirenda et al., 2018). Through phone-in programmes, radio can provide two-way communication information where the public can ask questions (WHO, 2020).

Our finding about the use of E-health in helping to address public health issues of concern is also consistent with findings from other studies (Fayoyin, 2016; Gayesa et al., 2023). However, some evidence points to the limitations of mHealth interventions and suggests that while the approach may be successful in reaching people, it could not be more effective in bringing desirable health outcomes (Bonvicini et al., 2022).

Our findings on the use of social media in public health interventions are also consistent with previous literature that suggest social media is a strategic source of epidemiological data and a massive platform for public health surveillance and response actions (Tambo et al., 2021). No wonder, in the COVID-19 RCCE, social media was an essential resource for getting community feedback and addressing the infodemic. The use of social media in RCCE, nevertheless, continues to attract caution as it also tends to exacerbate the spread of mis/disinformation and spurring infodemic and public anxiety (Ennab et al., 2022; Tangcharoensathien et al., 2020). Mis/disinformation, rumours and myths were some of the information-related challenges that affected Malawi’s COVID-19 RCCE response (Maganga et al., 2023).

For future outbreaks similar to COVID-19, we recommend the use of targeted training workshops as a key strategy. There is substantial evidence demonstrating the effectiveness of training in public health contexts. For instance, in China, training programmes significantly improved knowledge levels and enhanced attitudinal and behavioural intention scores among public health staff (Wang et al., 2008). In Iran, training was shown to increase the self-efficacy of emergency department nurses (Sheikhbardsiri et al., 2019), while in South Africa, emergency nurses reported improved knowledge and skills following training (Naidoo, 2017). Additionally, a systematic review focusing on training interventions in resource-limited settings found that such programmes can have a significant impact, although the effectiveness is highly influenced by the structure and design of the training (Van Lonkhuijzen et al., 2010). In the context of COVID-19 RCCE, training efforts could be particularly effective if directed towards community influencers, who can then leverage their positions to promote public health measures within their communities.

## Strengths and limitations

However, the current study had several limitations. The major limitation was that absence of community data that could triangulate results for this study. Further, the study collected data from only 10 participants and reviewed ten project reports only. The results may not be representative of all implementers of COVID-19 RCCE response. Additionally, due to COVID-19 restrictions, interviews for the study had to be done via telephone which limited the rapport and trust we made with the participants.

However, using a qualitative design, using high-level data from implementers and reviewing project reports helped to explore the subject in-depth, and to provide a localized perspective of approaches that can be used during emergencies.

## Conclusion

The current study’s findings have shown there are many approaches RCCE implementers in Malawi and even beyond can use in times of health hazards. However, choosing the most appropriate method is challenging, especially during infectious diseases like COVID-19, which change how people interact, and how the approaches are implemented. The study also shows that intrinsic characteristics of the system, urgency to deliver messages, accessibility and reach of the media and feasibility of the approach to be implemented during infectious disease are important factors to be considered. The study adds new literature that may inform the design and implementation of future RCCE responses in Malawi and other low-income countries where resources are minimal, and using the most appropriate channels help ensure available resources are put to best use. Future researchers may investigate the appropriateness of the approaches from a community perspective, which was limited in the current study’s scope.
